# The Protective Effect of Geraniol Against Hepatic Ischemia–Reperfusion Injury by Attenuating Oxidative Stress, Inflammatory Response, and Apoptosis in Rat Model

**DOI:** 10.1155/ijin/5571327

**Published:** 2025-10-12

**Authors:** Seyedeh Mahdieh Khoshnazar, Foruzan Delavarian, Sahar Rahimi, Shahriar Dabiri, Nader Shahrokhi, Nazgol Sharifi, Sara Shafieipour

**Affiliations:** ^1^Gastroenterology and Hepatology Research Center, Institute of Basic and Clinical Physiology Sciences, Kerman University of Medical Sciences, Kerman, Iran; ^2^Department of Internal Medicine, Kerman University of Medical Sciences, Kerman, Iran; ^3^Department of Physiology, Kerman University of Medical Sciences, Kerman, Iran; ^4^Pathology and Stem Cell Research Center, Kerman University of Medical Sciences, Kerman, Iran; ^5^Physiology Research Center, Institute of Neuropharmacology, Kerman University of Medical Sciences, Kerman, Iran

**Keywords:** geraniol, hepatic, ischemia–reperfusion, rat

## Abstract

**Background:**

Hepatic ischemia–reperfusion injury (HIRI) is one of the main causes of hepatic fibrosis that occurs during liver surgery. This study aimed to investigate the protective effect of geraniol (GNL) against HIRI in a rat model.

**Methods:**

Wistar rats were randomly divided into seven groups and subjected to 45 min of hepatic ischemia, followed by either 60 min or 6 h of reperfusion. Immediately before reperfusion, graded doses of geraniol (50 and 100 mg/kg) were administered intraperitoneally. Serum levels of alanine aminotransferase (ALT) and aspartate aminotransferase (AST) were measured to evaluate liver function. Antioxidant enzyme activities were assessed in liver homogenates. The concentrations of TNF-α, IL-1β, Bax, and Bcl2 mRNA and proteins in liver tissue were measured using RT-PCR and enzyme-linked immunosorbent assay (ELISA). The expression of Bcl2 and caspase-3 in liver tissue was evaluated by immunohistochemistry. In addition, liver tissue histopathology was examined under a light microscope.

**Results:**

The results demonstrated that liver damage significantly increased after repeated HIRI. However, treatment with GNL reduced hepatic enzyme levels and mitigated pathological changes resulting from repeated HIRI. Additionally, GNL treatment led to a decrease in apoptotic factors.

**Conclusion:**

GNL may be a potential therapeutic agent for preventing or treating hepatic fibrosis caused by ischemia–reperfusion injury.

## 1. Introduction

Hepatic ischemia–reperfusion injury (HIRI) is a common phenomenon that occurs in various clinical scenarios, such as liver resection, liver transplantation, shock, and trauma [1, 2]. HIRI causes tissue damage and organ dysfunction by inducing oxidative stress, inflammation, apoptosis, and necrosis responses [2, 3]. This complex cascade of events results in hepatocellular damage and activation of fibrogenic pathways [4]. The mechanisms of HIRI involve multiple cellular and molecular players, such as Kupffer cells, neutrophils, natural killer cells, endothelial cells, hepatocytes, cytokines, chemokines, reactive oxygen species (ROS), nitric oxide, and adenosine triphosphate [5]. HIRI also affects the regeneration and repair of the liver after injury [6]. Several strategies have been proposed to prevent or attenuate HIRI, such as pharmacological agents, ischemic preconditioning, ischemic postconditioning, and machine perfusion [7]. However, the clinical application of these strategies is limited by their efficacy, safety, and feasibility [8–14].

**Figure 1:**
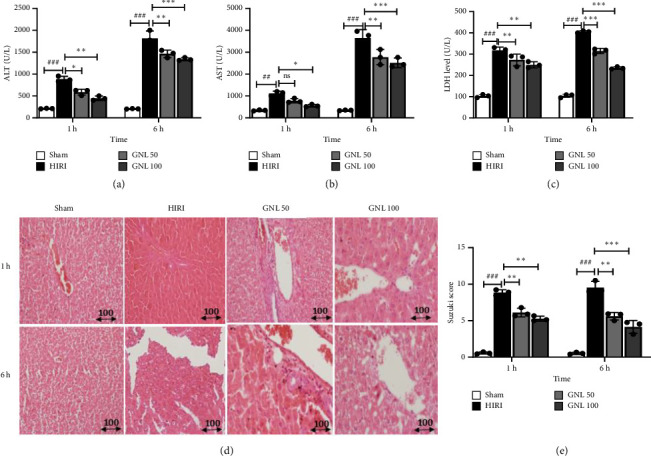
GNL ameliorated HIRI. The rats were randomly divided into the sham group, the HIRI group, and the HIRI + GNL (50, 100 mg/kg) groups. (a) Serum ALT, (b) AST, (c) and LDH levels in the sham group, HIR control group, and HIRI + GNL (50 and 100 mg/kg) groups. (d) Representative photomicrograph of liver histology with H&E staining in the sham group, HIR control group, and HIRI + GNL (50 and 100 mg/kg) groups at 1 and 6 h after reperfusion (magnification: × 200, scale bar = 100 μm). (e) Suzuki scores were presented in the sham group, HIRI control group, and HIRI + GNL (50 and 100 mg/kg) groups at 1 and 6 h after reperfusion. Data were presented as the mean ± SEM, *n* = 7/group. ^#^*p* < 0.05, ^##^*p* < 0.01, ^###^*p* < 0.001 vs sham group, ^∗^*p* < 0.05, ^∗∗^*p* < 0.01, ^∗∗∗^*p* < 0.001 vs HIRI group. HIRI, hepatic ischemia–reperfusion injury; H&E, hematoxylin and eosin; AST, aspartate aminotransferase; ALT, alanine aminotransferase; LDH, lactate dehydrogenase.

In recent years, there has been an increasing recognition of the therapeutic value of molecules derived from natural sources [15–17]. Furthermore, extensive research conducted over the past few decades has demonstrated that geraniol (GNL), a pure botanical compound, possesses diverse pharmacological activities by primarily modulating protein expression. Notably, this substance has received approval from the Food and Drug Administration (FDA) for its use as a flavoring and artificial supplement in human consumption [18–20]. The acyclic monoterpene geraniol, with the chemical formula (2E)-3,7-dimethylocta-2,6-dien-1-ol, is commonly present in the essential oils of numerous plant species (Figure 1). Plants such as lemongrass, rose, lavender, and other aromatic species contain significant amounts of geraniol. Research has demonstrated that GNL possesses a range of pharmacological properties, including antioxidant [21], antinociceptive [22], anti-inflammatory [23], antimicrobial [24], and antitumor activities [25]. These activities are attributed to geraniol's ability to regulate multiple signaling pathways involved in diverse biological processes [26, 27]. Thus, it is postulated that this particular compound, possessing a robust prophylactic capacity, can protect against oxidative and inflammatory alterations [28]. Additional scrutiny has revealed that GNL enhances the metabolic processes of inflammatory cells, augments the levels of GSH, and stimulates the activities of antioxidant enzymes [10]. However, during hepatic ischemia–reperfusion (I/R) injury, a severe inflammatory response and oxidative stress occur. These events exacerbate hepatocellular death and may result in liver dysfunction. Considering that hepatic ischemic injury is considered an acute disorder, it is expected that the onset of inflammatory reactions will be evident within the first hour and reach their maximum after 6 h. Therefore, with a profound understanding of the pathogenic mechanisms of I/R injury (IRI), various approaches, including antioxidants, have been developed for the prevention or treatment of IRI. This study represents the initial investigation to administer GNL via intraperitoneal injection immediately following reperfusion, a method that could produce different outcomes on the liver compared to pretreatment or delayed treatment. Intraperitoneal administration offers certain advantages over oral delivery, including enhanced bioavailability, faster absorption, and bypassing the gastrointestinal tract. In this study, we measured the levels of hepatic enzymes, inflammatory markers, oxidative stress indicators, and apoptotic factors in the serum and liver tissue of rats treated with GNL or vehicle before and after HIRI. We also performed the histopathological examination of the liver sections to assess the degree of tissue damage. Furthermore, the current investigation aims to provide a comprehensive understanding of GNL's potential as an anti-inflammatory and antioxidant agent, thereby paving the way for future experimental and clinical studies, as well as therapeutic applications.

## 2. Materials and Methods

### 2.1. Animals

A group of male Wistar rats (weighing 200–250 g) was purchased from the laboratory animal center at Kerman University of Medical Sciences and was kept in a standardized environment (with natural light/dark cycle, temperature of 23 ± 2 degrees Celsius, and humidity of 60 ± 5%). They were provided with free access to food and water. The study was conducted in accordance with the Basic & Clinical Pharmacology & Toxicology policy for experimental and clinical studies [29]. In this study, all animal experiments were conducted following the ARRIVE 2.0 guidelines, ensuring rigorous study design, reduction of bias, and transparent reporting.

### 2.2. Hepatic I/RI Model and Experiment Design

After a minimum of 7 days of acclimation, the rats were randomly divided into 7 groups: (1 and 2) control groups (HIRI), in which the rats underwent 1 h of ischemia followed by 1 h and 6 h of reperfusion. (3) Sham group: rats in this group underwent surgery but did not undergo IRI. (4 and 5) HIRI + GNL groups, in which the rats underwent 1 h of ischemia followed by 1 h of reperfusion, and immediately before the start of reperfusion, GNL was injected intraperitoneally with doses of 50 and 100 mg/kg. (6 and 7) HIRI + GNL groups, in which the rats underwent 1 h of ischemia followed by 6 h of reperfusion, and immediately before the start of reperfusion, GNL was injected intraperitoneally with doses of 50 and 100 mg/kg. First, the animals were anesthetized by intraperitoneal injection of ketamine (90 mg/kg) and midazolam (15 mg/kg). After preparing the surgical site, a 3-cm abdominal incision was made. The hepatic artery, portal vein, and bile duct were clamped to induce ischemia, which caused a change in the liver's color. Then, 0.5 mL of normal saline was poured over the animal's intestine, and the abdomen was sutured with 3-0 silk sutures. Hepatic samples and blood from the aorta were collected postreperfusion, and the rats were euthanized at 1 and 6 h with increased doses of anesthesia.

### 2.3. Biochemical Analysis

The blood samples underwent centrifugation at a force of 800 g for 15 min to prepare them for biochemical analysis. Following centrifugation, the resulting plasma was gathered and subjected to examination to determine the levels of SOD, GPx, CAT, as well as the enzymes alanine aminotransferase (ALT), aspartate aminotransferase (AST), and lactate dehydrogenase (LDH).

### 2.4. LDH Activity Assay

The LDH activity was assessed in a 20 μL aliquot of the cellular supernatant employing an LDH kit (Jian Cheng Bioengineering Institute, Nanjing, China) and a microplate reader (Biotek, USA).

### 2.5. Liver Function Evaluation

After anesthesia, 5 cc of blood was taken from the abdominal aorta of the animals. Then, the blood samples were centrifuged at 3500 rpm for 10 min, and the serum was collected. The separated serum was stored at a temperature of 20°C. The commercial kits (Asan Chemical Co., Cheonan, and Republic of Korea) were used to examine the liver enzymes ALT and AST [30, 31].

### 2.6. Measurement of Antioxidant Activity in Liver Tissue

To assess SOD, GPx, and CAT activities in liver tissue, washed tissue was homogenized (20%) and centrifuged at 11,000 rpm and 4°C for 10 min. SOD activity was measured calorimetrically at 440 nm using a Cayman kit, defining 1 U as a 50% reduction in superoxide radical. GPx activity was determined at 340 nm after initiating the reaction with cumene hydroperoxide. CAT activity was assessed by H_2_O_2_ consumption spectrophotometrically at 240 nm. Activities were expressed as U/mg protein, with protein quantified using the BCA assay and bovine serum albumin as a standard. All steps were done at 4°C, and homogenates were stored at −80°C before analysis [30].

### 2.7. Real-Time Polymerase Chain Reaction Analysis

The TRIzol reagent (Invitrogen) was employed to extract total RNA from frozen liver tissue. Then, the PrimeScript RT reagent kit (TaKaRa) was used to synthesize complementary DNA, following the instructions provided by the manufacturer. For RT-PCR, a total volume of 10 μL, consisting of SYBR Green PCR master mix (Applied Biosystems), was used. The amplification was carried out using the Light-Cycler 480 instrument (Roche Diagnostics, Penzberg, Germany) with the following cycling conditions: an initial denaturation step at 95°C for 5 min, followed by 45 cycles of denaturation at 95°C for 30 s, annealing at 55°C for 30 s, and extension at 72°C for 15 s. GAPDH (housekeeping gene) was used for normalizing data. The forward primer for rat TNF-α was 5′-GTCTCATCTCCGCCTTTGTC-3′; TNF-α reverse, 5′-ATGCCTTGCCTTTGAATCAC-3′; IL-1β forward, 5′-CTTATTGCCTCTGCCCTCTG-3′; IL-1β reverse, 5′-TGATTGGTCTGGACTGTGGA-3´; Bax forward, 5′-GTTCTGTCTCCCACCACCAT-3′ and reverse, 5′-GCCTGGTCTTGAACTTGCTC-3′ and Bcl-2 forward, 5′-GTGGTGGAGGACAGGAAGAG-3´; Bcl-2 reverse, 5′-CAGTGGTCTCCTGCTGAACA-3´; GAPDH forward, 5′-GAAAGGGACTTGGAGGAAGC-3´; GAPDH reverse 5′-CTTAAGAAGTGGCGGTTTGC-3´. A final extension step at 72°C for 5 min was performed. The RT-PCR results were analyzed using the 2^−ΔΔCt^ method, and each experiment was replicated three times.

### 2.8. Measurement of Hepatic Tissue Cytokines

The concentration of inflammatory mediators (TNF-α and IL-1β) in hepatic tissue was measured using an ELISA assay kit. The sandwich ELISA method was performed according to the manufacturer's protocol. The absorbance of the hepatic sample was measured using an AQ8 ELISA reader (Biotech). The levels of inflammatory mediators in tissue were ultimately determined in pg/mg of protein using a standard curve.

### 2.9. Measuring Apoptotic Factors

The levels of apoptotic factors including Bax and Bcl2 in liver tissue were measured using a sandwich ELISA method, according to the manufacturer's instructions (Sunlong Biotech, China). Homogenized liver tissue (20 mg per milliliter) was used, and the concentrations of Bax and Bcl2 proteins were assessed. The results were reported in picograms per milligram of total protein (pg/mg protein).

### 2.10. Histopathological Assessment of the Liver

Hematoxylin and eosin (H&E) staining was performed according to Bancroft and Gamble [32]. The quantitative analysis of the necrotic region seen in the H&E-stained sections was conducted with the assistance of Image J software. In histopathological studies, the severity of HIRI (inflammation, necrosis, sinusoidal congestion, and centrilobular ballooning) was blindly graded on a scale of 0–4 using modified Suzuki criteria: Grade 0: no damage, Grade 1: minimal damage, Grade 2: mild damage, Grade 3: moderate damage, and Grade 4: severe damage. All grading will be performed at 400x magnification and in 5 microscopic fields using an Olympus BX43 light microscope [33].

### 2.11. Immunohistochemistry Assay

Tissue expression levels of caspase-3 and Bcl-2 were assessed using immunohistochemistry. This method was performed using commercial kits (Abcam, Cambridge, MA, USA) according to the manufacturer's instructions. Sections fixed in formaldehyde were paraffin-embedded, baked at 60°C, deparaffinized, and rehydrated. After inactivating endogenous peroxidase with hydrogen peroxide and performing antigen retrieval in citrate buffer, sections were incubated with primary antibodies overnight at 4°C, followed by secondary antibodies. The staining was visualized using a light microscope (Olympus BX51, Tokyo, Japan) and scored semiquantitatively based on the percentage of positive cells. No positive cells (score: 0), mild staining, < 25% positive cells (score: 1), moderate staining, 25%–50% positive cells (score: 2), and severe staining, > 50% positive cells (score: 3).

### 2.12. Statistical Analysis

For the statistical analysis of the data, one-way analysis of variance (ANOVA) was used. In case of significant results, the Tukey test was employed to determine the level of significant differences between the groups. If the data distribution was non-normal, the nonparametric equivalent (Kruskal–Wallis and post hoc Mann–Whitney) was performed. All statistical analyses were conducted using GraphPad Prism 6. Furthermore, a significance level of *p* < 0.05 was considered, and the data in all figures were presented as the mean ± SEM.

## 3. Results

### 3.1. The Effect of GNL on Hepatic Function Evaluation

In the evaluation of liver cell damage, the effect of GNL was examined after HIRI. HIRI resulted in a significant increase in serum levels of ALT, AST, and LDH at 1 and 6 h after reperfusion compared to the sham group. However, in the HIRI + GNL 50 and 100 mg/kg groups, the serum levels of ALT, AST, and LDH at 1 and 6 h were significantly lower than those in the HIRI group. Notably, the serum ALT and AST levels in the HIRI + GNL 100 mg/kg group were significantly lower than those in the HIRI group (*p* < 0.001) (Figures 1(a) and 1(b)), and the serum LDH level in the HIRI + GNL 100 mg/kg group was also significantly lower than in the HIRI group (*p* < 0.01) (Figure 1(c)). Therefore, ALT, AST, and LDH levels were significantly reduced in the treatment groups, with a greater decrease observed in the high-dose group compared to the low-dose group. These results indicate that GNL can facilitate the improvement of liver function and the reduction of liver cell damage in a dose-dependent manner.

Liver injury in rats was evaluated using H&E staining. After 1 h of reperfusion, the sham group maintained normal hepatic structure, with slight central vein engorgement and open sinusoidal spaces. Hepatocytes appeared mostly normal. In contrast, the HIRI group showed increased congestion, vacuolization, and cell death. GNL treatment (50 and 100 mg/kg) led to hydropic degeneration in hepatocytes, evident from cytoplasmic vacuolations and hyperemic sinusoids, but without marked apoptotic or necrotic changes. After 6 h, the sham group showed less congestion and hyperemia, while the control group exhibited worsened hyperemia and cell death. GNL treatment reduced congestion and cell death. The Suzuki score confirmed GNL's protective effect against HIRI-induced liver damage, with significant improvements at both 1- and 6 h postreperfusion (*p* < 0.001) (Figure 1(e)).

### 3.2. The Effect of GNL on the Activity of Antioxidant Enzymes

**Figure 2:**
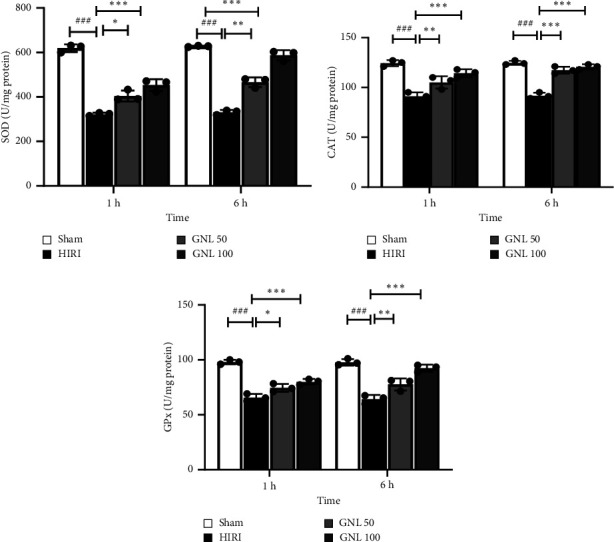
Effect of GNL on HIRI-induced oxidative stress. The tissue levels of GPx, CAT, and SOD were analyzed. Data were presented as the mean ± SEM, *n* = 7/group. ^#^*p* < 0.05, ^##^*p* < 0.01, ^###^*p* < 0.001 vs sham group, ^∗^*p* < 0.05, ^∗∗^*p* < 0.01, ^∗∗∗^*p* < 0.001 vs HIRI group. CAT, catalase; GPx, glutathione peroxidase; SOD, superoxide dismutase.

After 1 and 6 h following reperfusion, a remarkable decline was observed in the levels of crucial antioxidant enzymes, including superoxide dismutase (SOD), catalase, and glutathione peroxidase (GPx). This reduction in enzyme activity signifies the oxidative stress experienced by the liver tissue during the reperfusion phase. However, in stark contrast, the administration of an optimal dose of GNL (100 mg/kg) immediately after reperfusion in rats subjected to ischemia resulted in a significant increase in the activity levels of SOD, catalase, and GPx within the liver tissue compared to the control group (*p* < 0.05) (Figure 2). By boosting the activity of SOD, catalase, and GPx, GNL demonstrates its ability to scavenge harmful ROS and restore redox balance within the liver tissue.

### 3.3. The Effect of GNL on mRNA Expression of TNF-α, IL-1β

**Figure 3:**
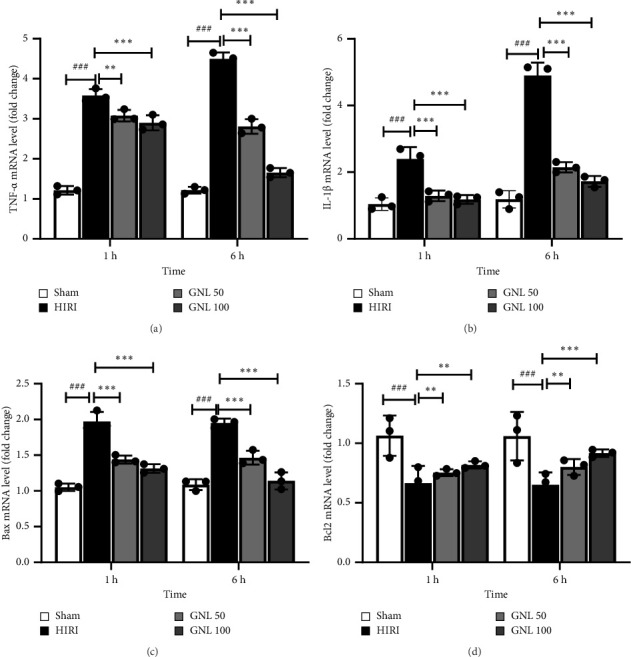
GNL reduced inflammatory and apoptosis responses induced by hepatic IR. Relative TNF-α (a), IL-1β (b), Bax (c), and Bcl-2 (d) levels were determined by RT-PCR. Data were presented as the mean ± SEM, *n* = 7/group. ^#^*p* < 0.05, ^##^*p* < 0.01, ^###^*p* < 0.001 vs sham group, ^∗^*p* < 0.05, ^∗∗^*p* < 0.01, ^∗∗∗^*p* < 0.001 vs HIRI group.

The results obtained from the reverse transcription-polymerase chain reaction (RT-PCR) assay revealed notable differences in the expression levels of proinflammatory cytokines, including TNF-α and IL-1β, at 1 and 6 h following reperfusion. In comparison to the sham group, the control group exhibited significantly elevated mRNA levels of TNF-α and IL-1β. However, a significant attenuation in the mRNA expression of TNF-α (*p* < 0.001) and IL-1β (*p* < 0.001) was observed in the liver tissue of rats receiving doses of GNL (50 and 100 mg/kg) following ischemia (Figures 3(a) and 3(b)).

### 3.4. The Effect of GNL on mRNA Expression of Apoptotic Genes (Bax and Bcl-2)

In the RT-PCR analysis, it was observed that subjecting rats to ischemic conditions had a profound impact on gene expression in the liver. Specifically, there was a noteworthy increase in Bax mRNA expression, while the expression of the Bcl-2 gene showed a significant decrease. However, when the rats were treated with GNL at a dosage of 100 mg/kg, a remarkable reduction in Bax mRNA levels in the liver was observed (*p* < 0.001) (Figure 3(c)). Additionally, GNL treatment led to a substantial increase in Bcl-2 gene expression (*p* < 0.01) (Figure 3(d)). These findings demonstrate that GNL administration effectively mitigates the upregulation of Bax mRNA and promotes the restoration of Bcl-2 gene expression in the liver under ischemic conditions.

### 3.5. The Effect of GNL on Protein Levels of TNF-α, IL-1β

**Figure 4:**
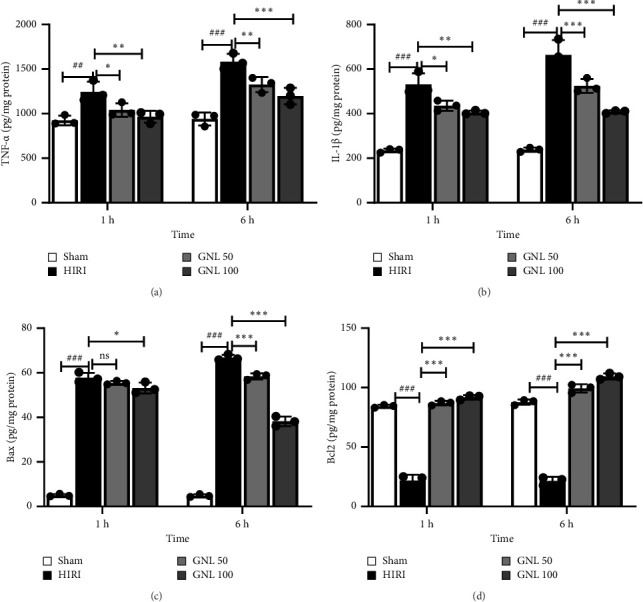
GNL reduced inflammatory and apoptosis responses induced by hepatic IR. Relative TNF-α (a), IL-1β (b), Bax (c), and Bcl-2 (d) levels were determined by ELISA. Data were presented as the mean ± SEM, *n* = 7/group. ^#^*p* < 0.05, ^##^*p* < 0.01, ^###^*p* < 0.001 vs sham group, ^∗^*p* < 0.05, ^∗∗^*p* < 0.01, ^∗∗∗^*p* < 0.001 vs HIR group.

Analysis through the ELISA test revealed compelling findings regarding the levels of TNF-α and IL-1β in liver tissue after reperfusion. At both 1 and 6 h following reperfusion, the control rats exhibited significantly higher levels of TNF-α, IL-1β, and IL-6 compared to the sham group (*p* < 0.001). On the other hand, in the group treated with GNL at a dose of 100 mg/kg following reperfusion, a remarkable reduction in the tissue levels of TNF-α and IL-1β was observed (*p* < 0.01 for both) (Figures 4(a) and 4(b)). This implies that the administration of GNL effectively mitigates the inflammatory response in the liver, as evidenced by the significant decrease in the levels of these proinflammatory cytokines.

### 3.6. The Effect of GNL on Protein Levels of Bax and Bcl-2

Upon close examination of the ELISA test results, it was observed that following 1 and 6 h of reperfusion, the control group displayed a remarkable increase in protein levels of Bax, while concurrently experiencing a significant decrease in protein levels of Bcl-2, compared to the sham group (*p* < 0.001). Conversely, the treatment group that received GNL at a dosage of 100 mg/kg exhibited a remarkable reversal in protein levels. Specifically, there was a significant decrease in protein levels of Bax, indicative of reduced apoptotic activity, and a remarkable increase in protein levels of Bcl-2, suggesting enhanced antiapoptotic defense mechanisms (*p* < 0.001) (Figures 4(c) and 4(d)).

### 3.7. The Effect of GNL on Apoptosis Response

**Figure 5:**
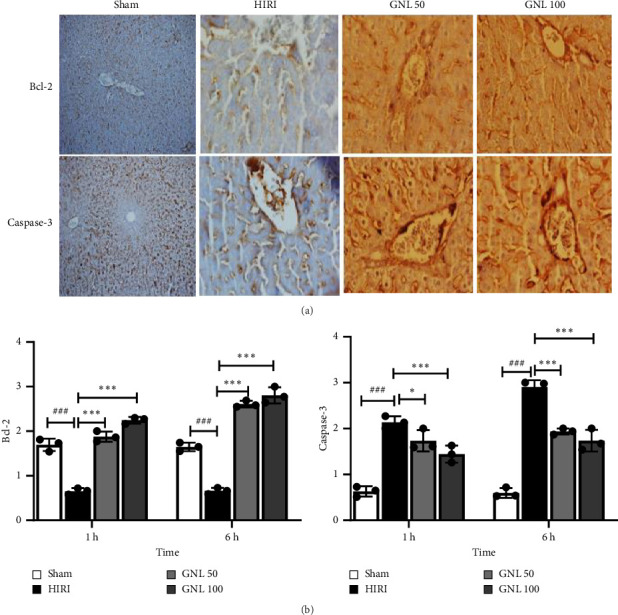
Immunohistochemistry was used to detect the expression of Bcl-2 and caspase-3 in liver tissue. (a) Immunohistochemical staining of Bcl-2 and caspase-3 in the sham group, HIRI control group, and HIRI + GNL (50 and 100 mg/kg) groups at 6 h after reperfusion (magnification: × 400, scale bar = 50 μm). (b) Relative positive expression analysis of Bcl-2 and caspase-3. Stained cells were scored as semiquantitative: no staining (score: 0), mild staining (score: 1), moderate staining (score: 2), and severe staining (score: 3). Quantified values were the mean ± SEM of at least three independent experiments. Data were presented as the mean ± SEM, *n* = 7/group. ^#^*p* < 0.05, ^##^*p* < 0.01, and ^###^*p* < 0.001 vs sham group, ^∗^*p* < 0.05, ^∗∗^*p* < 0.01, and ^∗∗∗^*p* < 0.001 vs HIRI group.

In the context of hepatocellular I/R, the expression of Bcl-2 and caspase-3 was notably prevalent. There were statistically significant differences in the scored caspase-3 and Bcl-2 values in liver tissue samples between the HIRI group and the HIRI + GNL group after 1 and 6 h of reperfusion (*p* < 0.001) (Figures 5(a) and 5(b)). Specifically, strong expression was observed in hepatocytes within the HIRI group compared to the sham group. Interestingly, there was no detectable Bcl-2 or caspase-3 immunohistochemical staining in the liver of the sham group after 6 h of reperfusion. However, positive Bcl-2 immunohistochemical staining was evident in Kupffer cells and hepatocytes within the HIRI group. Upon treatment with GNL, the expression of Bcl-2 in hepatocytes and Kupffer cells decreased relative to the HIRI group. Additionally, both the severity of immunohistochemical staining and the number of stained cells decreased in the GNL-treated group. Notably, numerous hepatocytes and sinusoids exhibited caspase-3 expression in the HIRI group, whereas caspase-3 expression was significantly lower in the GNL treatment group (Figures 5(a) and 5(b)). Statistical evaluation of the expression values for Bcl-2 and caspase-3 revealed a significant decrease in the GNL-treated group, whereas expression had increased in the HIRI group. These findings were positively correlated with the microscopic observations (Figure 1(e)).

## 4. Discussion

Reperfusion after a period of reduced blood supply to the liver can worsen liver damage during transplantation and other surgeries. Despite previous efforts, there are limited effective treatments available in medical practice for this condition. However, our recent study suggests that GNL has the potential to protect the liver from oxidative stress, inflammation-related injury, and hepatocyte apoptosis during I/R. Khoshnazar et al. recently emphasized the safety and efficacy of GNL in the digestive system, including the liver, and demonstrated its positive effects on digestive disorders. Geraniol has been shown to possess anti-inflammatory and antioxidant properties, which help reduce symptoms associated with various digestive disorders, including colon cancer, inflammatory bowel disease, irritable bowel syndrome, nonalcoholic fatty liver disease, liver fibrosis, and liver malignancies [34].

The species used for experimental investigation of hepatic IRI range from mice to pigs. Small animals such as mice and rats are exceptionally useful because they are easy to manage, present minimal logistical, financial, or ethical problems, and provide the potential for genetic alterations (e.g., transgenic and knock-out animals) [35]. In Huang's study, they employed the mice model for hepatic IRI [36, 37]. However, an important drawback is that the results of studies performed in small animals are of limited applicability to human beings due to their varying size and anatomy of the liver and their faster metabolism [35]. Nevertheless, we acknowledge that differences in immune responses and molecular pathways between species may influence the outcomes, and future studies using mouse models or complementary approaches will be valuable to further validate our findings.

Liver damage markers, such as ALT, AST, and LDH, play a crucial role in the pathophysiology of liver ischemia. These liver enzymes help identify liver damage or disease. AST is synthesized by a variety of tissues, including skeletal muscle, cardiac muscle, lungs, brain, kidneys, pancreas, erythrocytes, and leukocytes. Therefore, its elevated levels are not exclusively linked to liver damage, particularly in critically ill patients with compromised tissue perfusion. In contrast, ALT is predominantly found in the liver, and normal serum ALT levels can effectively rule out primary hepatic injury [38]. LDH is synthesized in response to hepatic impairment, and its levels are significantly elevated in liver injury due to hypoxic conditions, serving as an alternative pathway for ATP generation when cellular ATP supply is inadequate. Elevated AST, ALT, and LDH levels often indicate hepatic impairment within 12–24 h following an ischemic event. After addressing the primary pathology, AST levels return to the baseline more rapidly than ALT [39]. Our findings showed that serum levels of ALT, AST, and LDH significantly increased 1 and 6 h after reperfusion compared to sham groups (*p* < 0.05). GNL treatment reduced the activity of ALT, AST, and LDH, suggesting protective effects against parenchymal liver damage, consistent with Andrade et al.'s findings [40]. A subsequent study indicated that GNL ameliorated acute liver failure (ALF) in mice by reducing liver tissue pathology and inhibiting ALT and AST [41], supporting our current findings.

GNL's beneficial effects are attributed to its antioxidant properties and reduction of oxidative stress. Oxidative stress, caused by free radicals during ischemic events, leads to tissue injury by activating inflammatory pathways. Previous studies identified oxidative stress as a key mechanism in hepatic ischemic injury [42]. Our results confirmed that liver ischemia increases oxidative stress, triggering cytokine production, apoptosis, and leukocyte infiltration. A critical factor in antioxidant defense is the reduction of SOD activity, a sensitive indicator of hepatic cell damage. SOD and CAT enzymes protect against the harmful effects of H_2_O_2_ lipid peroxidation induced by oxidative stress. SOD converts superoxide into H_2_O_2_, while catalase and GPx detoxify H_2_O_2_ into water and oxygen [43]. In our study, SOD levels significantly decreased in rats with liver ischemia, leading to reduced GPX and catalase activity. GNL treatment preserved antioxidant enzyme levels, preventing the decrease in SOD and catalase while increasing GPX. This suggests that GNL enhances glutathione reductase, which reactivates glutathione, promoting detoxification. These results align with studies showing GNL's role in liver regeneration through its strong antioxidant properties [44]. Research has shown that a combination of terpenoids called caffeine and GNL can play a protective role in preventing Nitoside-induced mitochondrial damage, which causes cell death in hepatotoxicity [45]. A study investigated the extent of fibrosis in the liver, heart, and aorta of atherogenic hamsters treated with GNL. The previous findings of this study show that GNL reduces oxidative stress and maintains the status of enzymatic antioxidants under normal conditions. It has also been reported that GNL is a potent antioxidant that can scavenge free radicals produced by an atherogenic diet. This study demonstrated that supplementation with GNL reduces the expression of NF-κB and, consequently, oxidative stress. The findings of this study determined that the preventive action of GNL against fibrosis is primarily due to its antioxidant and anti-inflammatory properties through NF-κB signaling [23]. GNL's effects may be dose-dependent and interact with detoxification enzymes like GST, which plays a detoxifying and antioxidant role. Recent studies have shown that GNL inhibits GST activity in nematodes and rat liver, suggesting that its antioxidant capacity is influenced by enzyme interactions [46]. Research demonstrated that GNL mitigated I/R damage by attenuating oxidative stress through the Nrf-2/HO-1 pathway, reducing hepatic congestion in rats [47]. Zhou's investigation aligns with the current research and has demonstrated that GNL exhibits hepatoprotective properties against liver injury induced by bisphenol A. Its administration markedly ameliorates hepatic damage by enhancing the activities of SOD and catalase enzymes while concurrently diminishing malondialdehyde concentrations [48].

In hepatic IRI, inflammation mediated by proinflammatory cytokines plays a pivotal role [49]. Oxidative stress activates macrophages, which release cytokines and neutrophils. TNF-α and IL-1β, primarily produced by macrophages, amplify the inflammatory cascade [50]. Our study observed elevated TNF-α and IL-1β levels in the I/R group, indicative of inflammatory mediator release. GNL treatment significantly reduced these markers, corroborating its anti-inflammatory properties, in line with Ijaz et al.'s findings on GNL's cytokine modulation [43]. Similar anti-inflammatory effects of geraniol were noted in renal ischemia studies, affecting TNF-α, IL-1β, IL-6, and IL-10 levels [51]. It is noteworthy that a recent investigation has indicated that GNL possesses protective capacities against fulminant hepatic failure by upregulating PPAR-γ. PPAR-γ is a nuclear receptor that is integral to the regulation of adipogenesis, glucose homeostasis, and inflammatory responses. This receptor is extensively expressed across a variety of tissues, particularly in the liver, and is critical in the modulation of hepatic lipid metabolism and inflammation. Research has demonstrated that PPAR-γ has a protective function in ALF. In experimental models of ALF, PPAR-γ agonists have been observed to mitigate liver injury and inflammation while enhancing liver functionality [41]. Another study investigating the chemoprotective effect of GOH in cyclophosphamide-induced hepatotoxicity reported that GOH reduced oxidative and nitrative stress. It also showed anti-inflammatory activity by increasing PPAR-γ protein content and inhibiting p38 MAPK and JNK activation. In contrast, GOH-treated rats showed inhibition of P38 MAPK and JNK activation in a dose-dependent manner, which supports the anti-inflammatory effect of GOH [52]. GNL's anti-inflammatory properties also suppressed TNF-α, iNOS, and COX-2 levels in hepatic IRI [47]. Zhou's study has shown that GNL reduces the expression level of proinflammatory cytokines, namely, TNF-α, IL-1β, and IL-6, in liver injury, which is consistent with our studies [48].

In hepatic I/R, necrosis arises from ATP depletion, while apoptosis is ATP-dependent, initiated by death ligand/receptor interactions that activate caspases [53]. The cell's fate hinges on the balance of proapoptotic and antiapoptotic factors [54]. Apoptosis, often occurring late in I/R, was assessed in our study, revealing that GNL treatment increased Bcl-2 and decreased Bax mRNA and protein levels, paralleling decreases in caspase-3. Immunohistochemical analysis confirmed GNL's inhibition of caspase-3 activation and its promotion of Bcl-2's prosurvival role, which blocks subsequent caspase activation. Our findings align with Afolabi et al.'s emphasis on caspase-3 as an apoptosis marker in hepatic I/R [55]. El-Emam's study showed that GNL has antiapoptotic effects in liver tissue by reducing the level of Bax, caspase-3, and caspase-9 [47]. In the Zhou et al. study, GNL also reduced pyroptosis biomarkers (NLRP3, ASC, and caspase-1) in bisphenol A-induced liver injury [48]. The study indicates that GNL mitigates I/R-induced apoptosis by modulating apoptotic and antiapoptotic gene and protein expressions, suggesting a protective role for GNL in hepatic cell survival.

Recently, the concept of ischemia-free liver transplantation (IFLT) has gained considerable attention as a promising strategy to overcome the detrimental effects of IRI in liver grafts. Unlike conventional transplantation methods, IFLT aims to completely avoid ischemic insult by maintaining continuous oxygenated blood supply to the donor liver throughout the procurement, preservation, and implantation processes. According to a recent comprehensive review [56], IFLT has demonstrated potential to significantly reduce graft injury, improve early graft function, and enhance long-term outcomes. This progress underscores the critical importance of understanding molecular and cellular mechanisms underlying hepatic IRI. Integrating molecular insights with innovative surgical techniques like IFLT could pave the way for improved therapeutic strategies to mitigate liver IRI and enhance transplant success.

The histologic analysis revealed extensive hepatocellular necrosis and sinusoidal congestion in the hepatic tissue of rats subjected to I/R, indicating severe liver damage. However, treatment with GNL resulted in considerable areas of normal liver architecture and significantly reduced necrosis. This underscores the potential of GNL to protect against liver injury and preserve liver structure under I/R conditions. Several studies have investigated the effects of I/R on liver tissue and have reported similar findings. For example, Smith et al. conducted a study on mice and found that I/R resulted in hepatocellular necrosis and congestion, consistent with the observed histologic alterations in the present study. They also observed elevated levels of liver enzymes and inflammatory markers, indicating liver damage at a biochemical level [57]. Similarly, Roberts et al. examined liver tissue samples from patients who underwent liver transplantation, which involves I/R. They observed extensive necrosis and sinusoidal congestion, supporting the histologic findings mentioned in the statement. The authors also correlated these changes with increased oxidative stress and inflammation markers in the liver. Further research is needed to explore the underlying mechanisms and factors contributing to these observations and to determine the potential therapeutic effects of compounds like GNL in reducing liver damage during IRI [58].

## 5. Conclusion

Overall, the findings of this study suggest that GNL possesses beneficial effects on liver function and injury in the context of hepatic I/R. It exerts its protective effects through multiple mechanisms, including enhancing antioxidant defenses, attenuating inflammation, inhibiting apoptosis, and preserving liver structure. These results provide valuable insights into the therapeutic potential of GNL in mitigating hepatic injury associated with I/R. Further research is needed to elucidate the precise molecular pathways involved and to explore the significance of these findings.

## Data Availability

The data that support the findings of this study are available from the corresponding author upon reasonable request.
